# T66. PSYCHOMETRIC VALIDATION OF A NOVEL PATIENT-REPORTED OUTCOME MEASURE FOR ASSESSING PATIENTS’ SUBJECTIVE EXPERIENCE OF COGNITIVE IMPAIRMENT OF SCHIZOPHRENIA (PRECIS)

**DOI:** 10.1093/schbul/sby016.342

**Published:** 2018-04-01

**Authors:** Raymond Rosen, Jeremiah Trudeau, Steven Silverstein, David Henderson, Adam Smith, David Walling, Miguel Garcia, Bethany Davis, Leonard Derogatis, Michael Sand

**Affiliations:** 1 New England Research Institutes; 2 Boehringer Ingelheim Pharmaceuticals Inc; 3 Rutgers University; 4 Boston University School of Medicine, Boston Medical Center; 5 Richmond Behavioral Associates; 6 CNS Network, Inc; 7 Synexus; 8 Maryland Center for Sexual Health; 9 Boehringer Ingelheim Pharma GmbH & Co. KG

## Abstract

**Background:**

We have previously described the development and content validity of a new patient-reported outcome measure (PRO) to assess patients’ subjective experience of cognitive impairment in schizophrenia: The Patient-Reported Experience of Cognitive Impairment in Schizophrenia (PRECIS). Here we assess the psychometric properties of the PRECIS PRO in patients with schizophrenia and healthy age-matched controls with the aim of developing a revised version for use in clinical studies.

**Methods:**

The PRECIS PRO is a 35-item scale comprising eight concept domains (memory, communication, control, planning, handling problems, attention, sharp thinking and overall experience), each with multiple individual items. PRECIS was administered to psychiatrically-healthy controls (single visit), and a subset of patients in a large, clinical trial assessing patients with schizophrenia on stable antipsychotic treatment (NCT02281773) at baseline and Weeks 6, 9 and 12. Analysis of the original 35-item PRECIS PRO included factor structure, factor analysis (FA), internal consistency, test-retest reliability and discriminant (known groups) validity testing. FA was performed on all pre-treatment scores in the patient group (n=410) and patients and controls combined (n=498). Individual items with less than adequate reliability or validity were then identified and eliminated or modified.

**Results:**

Questionnaire responses were collected from 410 patients with schizophrenia and 88 healthy controls. The mean (standard deviation [SD]) total PRECIS score was significantly lower for healthy controls (1.39 [0.7]) compared with patients (2.06 [1.2]; p<0.0001), as was overall experience domain score (1.41 [0.7] vs 2.35 [1.3]; p<0.0001). For each domain of patient experience, PRECIS mean scores were also significantly lower for healthy controls compared to patients with schizophrenia. The mean differences between groups ranged from -0.94 (overall experience domain) to -0.52 (control domain; p<0.0001, all domains). Patients with schizophrenia had wider response distributions compared with controls, while the control group had marked “floor effects” across most items. Initial exploratory FA of the 35-item PRECIS PRO identified a 6-domain solution that accounted for 62% of total item variance, and Cronbach’s alpha (0.959) indicated an extremely high level of internal consistency. Following analyses of the 35-item PRECIS PRO, a total of 11 items were eliminated based on pre-specified criteria (poor loading onto identified factors, marked floor effects in patient groups or <50% test-retest reliability). Confirmatory FA of the revised 24-item PRECIS PRO identified 1 primary domain (attention) and 3 secondary additional domains (memory, executive function, communication). An additional domain included items related to patient distress or bother related to cognitive impairment. There was a high level of internal consistency both for the overall 24-item PRECIS PRO (Cronbach’s alpha= 0.942) and individual domains (Cronbach’s alphas: 0.743–0.873). Intraclass correlation coefficients were 0.78 for the overall 24-item PRECIS PRO and ranged from 0.49–0.74 for individual domains. Finally, discriminant validity testing confirmed there were significant differences between the patient group and the control group in each of the 5 domains of the revised 24-item PRECIS PRO (p<0.0001).

**Discussion:**

This large validation study demonstrated that the revised 24-item PRECIS PRO is a valid and reliable PRO measure with good internal consistency, adequate test-retest reliability and strong discriminant validity. PRECIS may therefore serve to define key patient-based endpoints for use in future clinical studies.

**Funding:**

Boehringer Ingelheim (1289.20)

